# Novel Weighting Method for Evaluating Forest Soil Fertility Index: A Structural Equation Model

**DOI:** 10.3390/plants12020410

**Published:** 2023-01-15

**Authors:** Wenfei Zhao, Xiaoyu Cao, Jiping Li, Zhengchang Xie, Yaping Sun, Yuanying Peng

**Affiliations:** 1 Faculty of Forestry, Central South University of Forestry and Technology, Changsha 410004, China; 2 Key Laboratory of State Forestry Administration on Forest Resources Management and Monitoring in Southern Area, Changsha 410004, China; 3 College of Arts and Sciences, Lewis University, Romeoville, IL 60446, USA

**Keywords:** age groups, soil depth, Chinese fir plantation, model, soil nutrients, soil fertility index

## Abstract

Understanding nutrient quantity and quality in forest soils is important for sustainable management of forest resources and maintaining forest ecosystem services. In this study, six soil nutrient indicators, including soil organic matter (SOM), total nitrogen (TN), total phosphorus (TP), available nitrogen (AN), available phosphorus (AP), and available potassium (AK) were measured in five different aged stands of Chinese fir forests in subtropical China. A structural equation model (SEM) was developed based on these soil nutrients indicators in order to better evaluate the soil fertility index (SFI) in these studied forests. The results show that soil nutrient contents changed with the soil depth in different age groups. The SOM decreased in a specific order: over mature > mature > near mature > middle > young stands. The TN content of the soil gradually decreased with increased soil depth throughout all age groups. The SEM indicated that the TN had the highest weight of 0.4154, while the TP had the lowest weight at 0.1991 for estimating the SFI. The weights of other indicators (AN, SOM, AP, and AK) ranged 0.2138–0.3855 in our study. The established SEM satisfied the fitness reference values and was able to accurately describe the forest soil nutrient status through the SFI. The overall SFI values were significantly higher in over mature stands than in young-aged stands and in topsoil than in deeper soil in all examined forests. Soil TN, AP, and AK were the most important nutrient indicators to the evaluation of the SFI in the study sites. The results confirmed that the SEM was suitable to estimate the weights of the SFI and better describe the soil nutrient status in forests. Our research provides an innovative approach to assess a soil nutrient status and soil fertility and provides a scientific basis for accurate implementation of soil nutrient assessment in forest ecosystems.

## 1. Introduction

Soil is an important component of forest ecosystems. Many important ecosystem processes occur in soil systems, such as nutrient uptake, decomposition, and water availability [1]. Soil nutrient status represents the soil’s ability to preserve environmental quality, ecological balance, and organism health [2]. Therefore, a comprehensive assessment of the soil nutrient status is very important for improving stand productivity and sustainable management of forest ecosystems [3].

Exploring soil nutrients status and soil fertility evaluation can provide scientific insight for forest management. The soil nutrient status is not only affected by independent effects of nutrient factors, but it also depends on the comprehensive coordination of various nutritional factors [4]. The evaluation of the soil nutrient status is mainly based on the comprehensive evaluation of nutrient factors in the study area [5].The soil fertility index (SFI) is a tool to comprehensively evaluate soil fertility, which is commonly used for soil fertility assessment and is critical for managing field variability and maximizing crop yields with minimal environmental impact [6]. It assures selection of the most appropriate properties of the soil, which have dominant influence on soil functions. Therefore, the SFI has been widely used in forests for assessing the soil nutrient status. In the estimation and evaluation of the SFI, a critical step is to determine the weightage of an individual indicator, which reflects the relative importance of each selected indicator in determining the soil function. Two approaches were commonly used to determine the weightage of soil indicators, namely an objective weighting approach and a subjective weighting approach. The objective weighting approach, including principal component analysis [7,8,9], rough set method [10], and Nemero index method [11], is based on the objective reality of the original data, but it highly relies upon the intrinsic relationships of the data and ignores the innate theoretical knowledge [12]. The subjective weighting approach was established based on hierarchical analysis and expert scoring methods [13], which are highly susceptible by human perception, but rarely consider the dependences of the interactions among soil nutrient factors [14]. Therefore, there is an urgent need to develop new methods for better assigning the weightage of soil nutrient parameters in forest ecosystems.

The structural equation modeling (SEM) is an extension of the general linear model (GLM) that combines a verification factorial analysis with a coupled equation system to verify multiple interrelationships between variables [15]. Modelling analysis includes validation analysis and exploratory analysis. Confirmatory factor analysis (CFA) uses the degree of fit test to determine the degree of fitness of the model and to conduct a comprehensive evaluation of the model avoiding the evaluation of direct relationships between individual variables [16]. Exploratory factor analysis (EFA) can simultaneously explore the relationships between multiple variables in the system and determine the impact intensity for comprehensively reflecting the overall state of soil nutrients and the complex influence relationships and mechanisms among various indicators [17,18,19]. The SEM can analyze the relationships between various factors in the system based on the existing theoretical knowledge to evaluate soil nutrient status. The path coefficients are calculated using the logical relationship of the data itself. This illustrates the theory of direct and indirect causality between the correlated variables [20,21].

Chinese fir (*Cunninghamia lanceolata* (Lamb.) Hook.) is one of the most important fast-growing timber species in the subtropical regions of China. Chinese fir plantations have more than 1000 years of cultivation history with high yield and excellent woody quality [22]. However, the large-scale planting of pure Chinese fir plantations has resulted in soil degradation, such as soil nutrient loss, increases pests and diseases, and decreases soil quality. These problems seriously affect stand growth and sustainable management in Chinese fir forests [23]. In order to improve the soil quality of Chinese fir plantations, the appropriate approach for assessing the soil nutrient status will provide a theoretical basis for sustainable management of Chinese fir forest ecosystems. The SEM has been previously used in the fields of psychology and medicine [24,25] and it was applied in forestry and soil science recently [19,21]. However, this method is rarely used in assessing the soil nutrient quality in forest soils.

The objectives of this study were: (1) to determine the variation of soil nutrients indicators in different aged stands of Chinese fir plantations with different soil depths; (2) to develop a SEM for evaluating the soil fertility index by weighting soil nutrients in the study forests; and (3) to assess the soil nutrient indicators of different aged stands with soil depth of Chinese fir plantations by using the SFI. 

## 2. Results

### 2.1. Soil Nutrient Characteristics

As shown in Table 1, the SOM content of each stand ranged from 13.07 to 18.15 g·kg^−1^, with the highest SOM content in age group V and the lowest in age group II. There were significant differences in SOM content between age groups (*p* < 0.05). The content of TN varied from 0.74 to 0.89 g·kg^−1^ and decreased in order: IV > V > III > I > II (Table 1). There was a significant difference in the TN content between age group II and age groups V and IV (*p* < 0.05). However, the soil TP, AN, AP, and AK contents had no significant differences among age groups (*p* > 0.05, Table 1).

**Figure 1:**
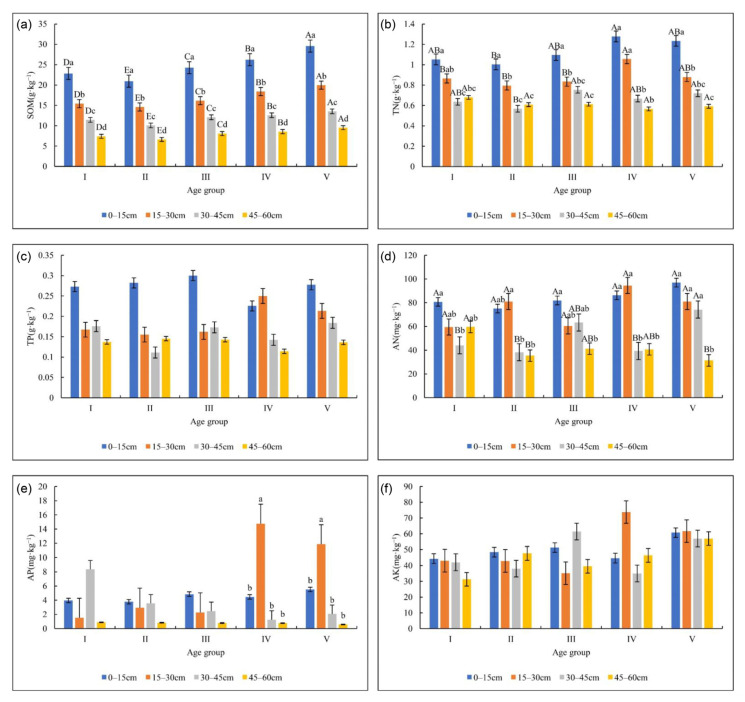
Soil nutrient contents change with soil depth in different age group stands. Note: (**a**) represents SOM, (**b**) represents soil TN, (**c**) represents soil TP, (**d**) represents soil AN, (**e**) represents soil AP, and (**f**) represents soil AK in figure. Different upper-case letters in the graphs indicate significant differences between the same soil layers in different age groups (*p* < 0.05), while lower-case letters indicate significant differences between different soil layers in the same age group stand (*p* < 0.05) in the figure.

The soil nutrient contents changed with the soil depth in different age groups, as shown in Figure 1. The SOM content of all the stands decreased significantly (*p* < 0.05) as the soil depth increased. The content of the SOM decreased as V > IV > III > II > I among the age group stands (Figure 1a). The soil TN content gradually decreased with increased soil depth throughout all age groups (Figure 1b). The soil TN contents were higher in stands III, IV, and V than in stands I and II, and were significantly different among the soil layers with a decreasing order: ranked topsoil (0–15 cm) > soil layer (15–30 cm) > (30–45 cm) > (45–60 cm) in group age stand V, and topsoil (0–15 cm) = soil layer (15–30 cm) > (30–45 cm and 45–60 cm) in age groups II, III, and IV stands, respectively (Figure 1b). The significant differences of the soil TN contents were found among three soil layers in age group I (Figure 1b).

There were no significant differences (*p*> 0.05) of soil TP and AN contents among the age groups (Figure 1c,d). The TP content decreased as the soil depth increased in age group V (Figure 1c), while the rank of soil TP was found in topsoil (0–15 cm, 15–30 cm) > 30–45 cm and 45–60 cm in age groups of II, IV, and V (Figure 1c). The soil AN was constantly higher in the topsoil layers (0–15 cm and 15–30 cm) throughout all age groups (Figure 1d). The soil AP content had no significant difference among age groups (Figure 1e), but it was significantly higher in the soil layer at 15–30 cm than in other soil layers in age group IV and V, while there were no significant differences among the soil layers in age groups I, II, and III stands (Figure 1e).

The soil AK content had no significant differences among the soil layers throughout all age group stands, excepting a higher soil AK content was found in the layer of 15–30 cm in age group IV (Figure 1f). 

### 2.2. Weighting of Indicators

The reliability test of Cronbach’s alpha value was 0.829, indicating highly reliable data. The validity test of the KMO value was 0.750, which was greater than the threshold value of 0.7 (Table 2). In addition, Bartlett’s test of *p*-value was 0.000, indicating the data were very suitable for factor analysis. 

The SEM was modified from the MI calculated using AMOS 24.0. After modification, the ration of CMIN/DF was 2.844, the values of CFI, TLI, and IFI were 0.970, 0.911, and 0.971, respectively, while the value of SRMR was 0.0536 (Table 2). Therefore, all fit indexes had an excellent fit for the requirements of fit reference values and the degree of adaptation (Table 2). In addition, the CR of the model combination was 0.8717 with the AVE at 0.5505 and both values were greater than the critical value at 0.7 and 0.5, respectively [26], indicating the observed variables have strong internal correlations.

**Figure 2:**
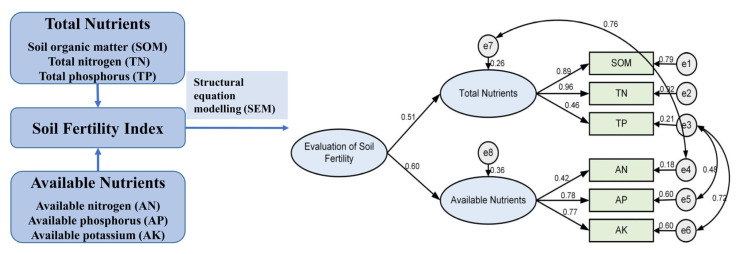
Modified model for the evaluation of soil fertility.

As shown in Figure 2, the path coefficients for the endogenous latent variables for the total and the available soil nutrients were 0.51 and 0.60, respectively. The path coefficients for the observed variables including SOM, TN, and TP to total nutrient indicators were 0.89, 0.96, and 0.46 (Figure 2), respectively. The path coefficients of the observed variables of soil AN, AP, and AK to the soil available nutrient indicators were 0.42, 0.78, and 0.77 (Figure 2), respectively. The standardized residual is a measure of the strength of the difference between the observed and expected values, which is repressed as a ratio. The SMC of the standardized residuals were 0.79, 0.92, 0.21, 0.18, 0.60, 0.60, 0.26, and 0.36 (Figure 2), respectively, in the present study.

The weights of the soil nutrient indicators were obtained by normalizing the above path coefficients (Table 3). The weights of the total nutrients (0.4604) and the available nutrients (0.5396) were not significantly different among the total nutrients. TN had the highest weight of 0.4154. It was a decisive factor affecting the total soil nutrients. TP had the lowest weight at 0.1991. The weight of the SOM was 0.3855. Among the available nutrients, AP and AK contents had similar weights at 0.3941 and 0.3921, respectively, while AN had the lowest weight at 0.2138.

### 2.3. Calculation of a Soil Fertility Index (SFI)

**Figure 3:**
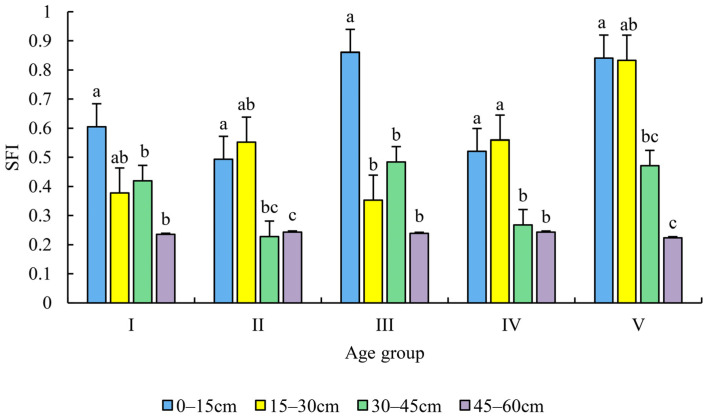
The soil fertility index (SFI) was evaluated in age groups and soil depth. Note: different lower-case letters indicate significant differences among different soil layers in the same age group (*p* < 0.05).

There was no significant difference in the SFI among the different age groups in the same soil layer (*p* > 0.05) (Figure 3). 

The age group I stand had the highest SFI in the 0–15 cm soil layer (0.61), which was significantly higher than the 30–45 cm soil layer (0.42) and 45–60 cm soil layer (0.24) (*p* < 0.05). The 15–30 cm soil layer had the highest SFI (0.55), which was significantly different from the 45–60 cm soil layer (0.24) (*p* < 0.05) in age group II (Figure 3). The SFI value of the 0–15 cm soil layer (0.49) was significantly higher than that of the 30–45 cm soil layer (0.23) and the 45–60 cm soil layer (0.24) (*p* < 0.05). In age group III, the SFI (0.86) was significantly higher in the 0–15 cm soil layer (0.86) than in the 15–30 cm (0.35), 30–45 cm (0.48), and 45–60 cm soil layers (0.24) in age group III (*p* < 0.05) (Figure 3). The SFI values for 0–15 cm (0.52) and 15–30 cm (0.56) was similar, and both were significantly higher than the 30–45 cm (0.27) and 45–60 cm soil layers (0.24) in age group IV (*p* < 0.05) (Figure 3). The SFI was similar in the 0–15 cm (0.84) and the 15–30 cm soil layers (0.83) in the age group V stand (Figure 3). The SFI was significantly higher in the 0–15 cm soil layer than in the 30–45 cm soil layer (0.47) and the 45–60 cm soil layer (0.22) (*p* < 0.05). The SFI was significantly higher in the 15–30 cm soil layer than in the 45–60 cm soil layer (*p* < 0.05) (Figure 3). Overall, the SFI value of the topsoil of all age group stands was at a high level, indicating that the topsoil was rich in nutrients, while the SFI value of the 45–60 cm soil was lower and relatively stable, indicating that the soil nutrient contents were significantly lower in the deeper soils (Figure 3).

## 3. Discussion

Soil quality and function are interrelated concepts that represent the range of soil properties and their associated ecological processes, which is fundamental to ensuring healthy forests [12]. The current study found that the soil SOM and the TN significantly differed across age groups. This was supported by the previous studies on different-aged Chinese fir plantations (10, 20, and 30 years), which demonstrated that the soil organic carbon and total nitrogen contents increased with increasing forest age [27,28]. The content of each nutrient indicator selected in this study was lower in the middle-aged stand soil and higher in the mature and overmature stands compared with other stand soils. This phenomenon is consistent with the changing pattern of nutrient uptake at different stages of tree growth [29]. For the middle-aged stands, trees are in the rapid growth stage and absorb large amounts of nutrients from the soil. The uptake of nutrients from soil is greater than the return nutrients to soil. In contrast, the trees were in a slow-growing stage in mature and overmature stands. The less demand for tree growth and less soil nutrients uptake by roots resulted in a higher content of nutrient in soils [30]. In addition, as stands become denser, soil nutrient returns increase, and soil nutrient requirements decrease [31]. The contents of soil SOM, TN, and AN were significantly affected by the soil depth and most of the nutrient indicators showed a high content of nutrients in the topsoil layer in the present study. The topsoil has most activities for soil fauna and microorganisms, and it is the main place for the internal circulation of soil nutrients by the plant apoplast [32,33]. Microbial activity transforms the dead woody debris and leaves that the organic matter accumulates on the soil surface to form humus [31]. Soil nutrients are absorbed by plant roots and returned to the soil through processes such as decomposition of litter, apoplastic matter, and roots turnover, so that the nutrient contents would be high in topsoil [34]. 

Soil provides essential nutrients for plant growth, and plants affect soil nutrients through litterfall, root growth, and exudates. This correlation also changes with stand age due to plant growth [35,36]. The content of SOM rose gradually as the forest of Chinese fir plantations aged, which was mainly affected by the aboveground litterfall and belowground root exudation by trees [37]. The needle litter of Chinese fir is difficult to decompose and might accumulate more SOM in the soil [38] However, the absorption of nutrients varied in different ages of Chinese fir plantations due to various growth rates and needle litters under over-mature and mature stands decomposing faster than the needle litters under near-mature and middle-aged fir forest stands [39], which could partly explain why there was no significant difference in soil TP, AN, AP, and AK contents in the studied forests. TN and AN demand and litter input of Chinese fir were clearly age-dependent and determined soil nutrient status because the rapid absorption and biomass accumulation in young stands depleted soil nutrients, whereas older stands had more litter returns and slower nutrients re-accumulation in the soil [40]. No significant differences of N were found between the stand ages in the Atlantic Forest [41] and the concentrations of all nutrients were statistically equal for all forest ages in mineral soil of riparian coniferous forests [42]. In addition, the results show that TP and AP contents were lower in our research, which could further explain the insignificant amount of P in different ages of stand soils in this study. This might be due to the warm temperature and the high precipitation of a mid-subtropical climate condition in the study region. The high precipitation accelerated the leaching of soil nutrients and resulted in the low phosphorus content of the soil [43]. Therefore, the loss of soil phosphorus has been a serious problem in mid-subtropic regions of China, and the loss of phosphorus was above the national average level [44,45]. In addition, the chemical interaction between phosphorus and clay minerals in mid-subtropical soils led to the formation of insoluble phosphorus by binding metal elements tightly in the soil, which is difficult for plants to absorb [46]. Soil heterogeneity occurs both horizontally and vertically, so the lack of standardized sampling procedures to assess SOC stocks may affect conclusions drawn from different studies [47].

In our study, the SEM combines path analysis, factor analysis, and multivariate analysis of variance. SEM, unlike other methods, does not separate single variables affecting the process from the system. It was built on prior knowledge of current theories and closely integrates classic multiple regression analysis of causation with factor analysis of latent variables. The SEM allows both overall evaluation of the model and measurement of the model fit through a fitness test and overcomes the effects of multicollinearity [48]. Latent variables that cannot be directly measured are estimated from the observed variables that can be directly measured or readily quantified, making the exploration of direct or indirect relationships between multiple variables of soil nutrients possible [49]. The weight of soil nutrients was determined according to the influence of the path coefficients. This approach provides a comprehensive and unbiased assessment of soil nutrient status. The results show that the developed SEM can fully reflect the relationships between soil nutrients in the study area. In this study, 500 sets of research data were used to build the model. The sample size is 10 times the indicators number and was able to meet the model construction needs [26].

The results indicate that the total nutrients and the available nutrients were equally weighted. The content of the total soil nutrients reflects the ability of the soil to supply nutrients, and the available nutrients reflect the intensity of the nutrient supply, both of which have important effects on the soil nutrient status of the forest stands. TN is the most demanded nutrient element influencing the total soil nutrient status during plant growth and affects the synthesis of plant proteins and nucleic acids. TN not only reflects the basal fertility of soil nitrogen, but also reflects the potential fertility of the soil. It has a significant impact on the vegetation composition and function of terrestrial ecosystems [50,51,52]. Our results are consistent with observations from another study [53,54]. Soil AP and AK had the greatest weight among the available nutrient indicators in our study. Soil phosphorus and potassium levels depend on source material, degree of weathering and leaching [34]. Southern China has a subtropical climate, and the soil types are predominantly red soil and yellow soil with richness in minerals such as alumina and iron oxide [55]. Iron and alumina are important minerals in soil because they easily combine with phosphorus to form insoluble phosphorus that plants have difficulty absorbing. In addition, Chinese fir plantations are coniferous forests, and coniferous litter contains a lot of refractory substances, such as tannins, waxes, and resins, and the high contents of lignin limits the recovery rate of nutrients [55,56]. Therefore, AP shortage in forests has been an important factor limiting the yield of plantations, and phosphorus-deficient plants may mature at a slower rate than plants with adequate amounts of phosphorus [54].

Our study revealed a high level of SFI (0.35–0.86) in the topsoil throughout all age groups stands, indicating that the topsoil of the study sites was not considered nutrient-deficient. This is similar to the results produced for the single indicator analysis. In contrast, the SFI (0.22–0.24) values were constantly lower in deeper soil layers (45–60 cm) throughout age groups stands. This may be due to the difficulty for plant roots to penetrate deep into the soil, as well as fewer activities of animals and microorganisms in deeper soil layers [57]. Overall, the soil nutrient status in the study stands was at the average level, with most of the SFI values < 0.6. Therefore, thinning with multi-species planting forest management should be considered in Chinese fir plantation stands because the complex and diverse root architecture and ground apomixis would improve soil nutrient availability and soil structure and texture [54].

## 4. Materials and Methods

### 4.1. Study Area

**Figure 4:**
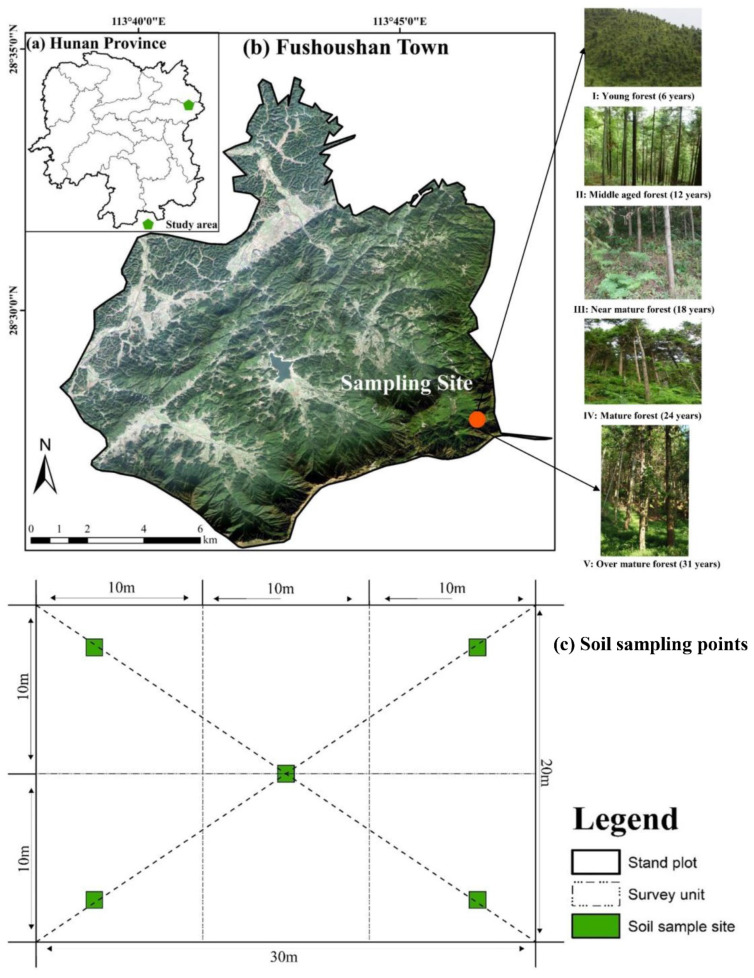
Geographical location of the study area in Fushoushan Town, Pingjiang County, Hunan Province, China. (**a**) The geography of Hunan Province, (**b**) the geography of Fushoushan Town, and (**c**) five soil sampling points evenly distributed in each plot of age group forest.

The study area was in Fushoushan Town, Pingjiang County, Hunan Province of China (28°25′00″–28°32′30″ N, 113°41′15″–113°48′45″ E) (Figure 4). The total area of the forests is 1134 ha including the public welfare forests of about 914 ha. The landform is hills with elevation of 835–1573 m. The slopes of the study area ranged 22–37°. The climate of study site is a typical subtropical humid monsoon with an annual average temperature and precipitation of 12.1 °C and 2100 mm, respectively. The soil is mountainous yellow loam developed from metamorphic shale and sandstone with high weathering degree and is slightly acidic with soil pH 4.57. The study area was covered by subtropical evergreen broad-leaved forests historically, but it was replaced by Chinese fir plantations after forest management [58]. The most dominant tree species in overstory is Chinese fir (*Cunninghamia lanceolata* (Lamb.) Hook.) with sporadic distribution of Cathay hickory (*Carya cathayensis* Sarg.) and sassafras (*Sassafras tzumu*). The understory shrubs are rhododendron (*Rhododendronsimsii* Planch), Chinese sumac (*Rhus chinensis* Mill.), bush clover (*Lespedeza bicolor* Turcz.), and holly (*Ilex chinensis* Sims). The ferns (*Pteridophyta*), Japanese silver grass (*Miscanthus floridulus*), Houttuynia cordata (*Houttuynia cordata* Thunb.), and Polygonatum (*Polygonatum sibiricum*) are the dominant herbaceous species in the understory.

### 4.2. Experimental Design and Data Collection

This study was conducted in 2018–2019. A split-plot design was used with the stand age as the main factor and the soil depth as the subfactor. In the study sites, 5 age groups of Chinese fir plantations with similar site conditions were selected, and they were young (6-year-old, as age group I), middle-aged (12-year-old, as age group II), near-mature (18-year-old, as age group III), mature (24-year-old, as age group IV), and over-mature (31-year-old, as age group V) stands. The five groups (group V to group I) of Chinese fir plantations were established in 1987, 1994, 2000, 2006, and 2012, respectively. Five 20 m × 30 m plots were set up for each of the age groups of Chinese fir stands. Thus, a total of 25 plots were established in the study site. The diameters at the breast height (DBH) > 2.0 cm and tree heights of all Chinese fir within the plots were numbered and measured (see Table 4).

Five soil sampling points were taken along two diagonal lines of each replicated plot in each age group (see Figure 4c). Four soil samples were collected from 0–15 cm, 15–30 cm, 30–45 cm, and 45–60 cm soil layers using a 4.5 cm inner diameter soil auger at each soil profile. Thus, a total of 500 soil samples (5 forest age groups × 5 replicating plots × 5 sampling points × 4 soil depths) were collected in the present study. The soil sample was sealed in a clean plastic bag and brought back into the lab for further treatment and analysis. The environmental characteristics of the stand plots and the sampling sites in each Chinese fir stand are shown in Table 4.

Six soil nutrient indicators, including soil organic matter (SOM), total nitrogen (TN), total phosphorus (TP), available nitrogen (AN), available phosphorus (AP), and available potassium (AK) were selected as soil nutrient indicators in the current study because they are appropriate soil fertility indexes and can reflect and represent the forest soil nutrient status adequately [14]. The content of the SOM was determined by using the potassium dichromate oxidation method [59]. The TN content was measured by using the Kjeldahl method [60]. The TP content was measured by using the sodium hydroxide alkaline solution molybdenum antimony anti-colorimetric method [61]. The AN content was determined by using the alkaline hydrolysis diffusion method [62]. The AP content was measured by using the sodium bicarbonate leaching-molybdenum antimony anti-colorimetric method [62]. The AK content was measured by using the ammonium acetate leaching-flame photometric method [63].

### 4.3. Variable Selection and Model Development

The structural equation model (SEM) is a multivariate method of statistical analysis used for building, estimating, and testing causal models. It includes regression analysis, factor analysis, path analysis, and multivariate analysis of variance, which is divided into measurement models and structural models. The formulae are as follows [64]:

(1) $$X=\Lambda_{x}\xi +\delta$$

(2) $$Y=\Lambda_{y}\eta +\varepsilon$$

(3) η=Bη+Γξ+ζ

Equations (1) and (2) are measurement models, where X is the measurement variable for ξ, Y is the measurement variable for η, ξ is the exogenous latent variable, η is the endogenous latent variable, δ,ε are the measurement error vectors, and $\Lambda_{x},\Lambda_{y}$ are the correlation coefficient matrices for the measurement variables X,Y and the latent variables ξ, η. Equation (3) is a structural model that describes the latent variables’ causal connection, where B represents the correlation coefficient matrix among the endogenous latent variables, Γ represents the influence of exogenous latent variables ξ on endogenous latent variables η, and ζ represents the unexplained part of the model, which is the error in endogenous latent variables. 

In this study, we selected six nutrient variables including two endogenous latent variables (soil total nutrients and the available nutrients) and one exogenous latent variable (the evaluation data of the soil fertility index) to build the SEM. In SEMs, the latent variables are often described as ellipses. Rectangles or squares are used to represent the observed variables. The error variables are the part of the SEM that cannot be effectively explained by the endogenous latent variables. It is the unique variables that cannot be explained by other variables, which are represented by a circle in the model. The causal link between the effects is illustrated by the direction of the arrows connecting the variables [21,65].

First, Cronbach’s alpha was calculated for data reliability analysis using SPSS 25.0 [66]. The data reliability is high when Cronbach’s alpha > 0.8; if 0.5 < Cronbach’s alpha < 0.8, the data reliability is acceptable; if Cronbach’s alpha < 0.5, the data reliability is not acceptable, and then the Kaiser–Meyer–Olkin (KMO) criterion and Bartlett’s test of sphericity are calculated [67]. When KMO > 0.7, it means that there is a common factor between the variables, which is suitable for factor analysis; if KMO > 0.5, it means that the data have a good and significant correlation, and the validity of the data test is good. When Bartlett’s spherical test result *p*–value < 0.05 (that is, *p* < 0.05), it indicates that the data are spherically distributed and there is a certain degree of independence between variables [68].

Second, using AMOS 24.0, a theoretical model for evaluating the soil fertility index was established based on prior theoretical knowledge and early experimental results (Figure 2). Data from five hundred soil samples were used for building the structural equation model (SEM). Normalization data was performed using the Z-score method available in SPSS 25.0. The normalized sample data was then fed into AMOS 24.0 for identification of the theoretical models described above. 

A test of fit evaluates a measurement model used to determine whether the data fit the hypothesis. The types of model fitting include absolute fitting, parsimonious fitting, and incremental fitting [16]. In this study, we used a standardized chi-square index (CMIN/DF; where CMIN = chi-square value, DF = degrees of freedom), a standardized root mean square residual error (SRMR), a comparative fit index (CFI), an unstandardized fit index (NNFI) (a.k.a. Tucker–Lewis Index, TLI), and an incremental fit index (IFI) for model fit testing using AMOS 24.0 output [69,70,71]. The reference value of CMIN/DF is in the range of 1–3. SRMR is required to be less than 0.08. CFI, TLI, and IFI are required to be less than 0.9. The model’s intrinsic fitness test statistics included combined reliability (CR) and average variance extracted (AVE). The reference values of CR and AVE are required to be greater than 0.7 and 0.5, respectively [26]. After the model is estimated and tested for fit, if the model does not fit well, it is necessary to perform model correction on the model that does not fit or whose indicators are unreasonable. According to existing research, the correction methods can include adding or deleting variables and changing the paths. In addition, some scholars have modified the model according to the modification index (MI) indicator of the AMOS output report. This method performs the above model estimation and testing steps again on the revised model until a model that both conforms to the theoretical assumptions and has a good fit is obtained [67]. This study modified the model based on the MI indexes of the AMOS 24.0 output report.

### 4.4. Methods for Evaluating Soil Nutrient Status

The structural equation model (SEM) results are used to calculate the soil nutrient weights. The formula for the SEM is as follows [26]:

(4) $$W=\sum_{h=1}^{H}\beta_{h}[\sum_{h=1}^{H}\rho_{k}m(h,k)]$$

where m(h,k) represents the score of the indicator *k* in the large category *h*, $\rho_{k}$ represents the weight of the indicator *k*, and $\beta_{h}$ represents the weight of the large category *h*.

In order to make each soil parameter comparable across multiple units, the data were made dimensionless using scoring function equations. After processing, the numerical range is converted to a value between 0 and 1, where 1 indicates a higher indicator value (e.g., concentration) and 0 indicates a lower indicator value [72]. Non-linear scoring methods have been found to be more discriminative than linear scoring methods [73]. Therefore, we used a non-linear scoring equation to calculate the soil indicator scores in our study [Equation (5)].

The soil nutrient parameters were assigned to one of two groups based on their effects on the soil nutrient status. When an increase in the number of indicators improved soil quality, the “more is better” scoring curve was used. Conversely, a ‘less is better’ scoring curve was used when increases in the soil parameter levels were deemed detrimental to the soil quality. The indicators selected for this study were ‘the more the better’. The nonlinear score (*S_i_*) of the soil indicators (possible values from 0 to 1) could be expressed as follows [74]:

(5) Si=11+(xx0)b

where *x* is the actual measured value of each metric, *x_0_* is the average value of each metric, *b* is the slope of the equation, the “bigger is better” metric has a value of −2.5, and the “smaller is better” metric has a value of 2.5 better indicator [74].

After weighing and scoring all soil fertility indexes (SFI), the SFI was determined. The formula is expressed as follows [75]:

(6) $$SFI=\sum_{i=1}^{n}W_{i}\times S_{i}$$

where *SFI* is the soil fertility index, *S_i_* is the index score, *W_i_* is the weighting of the index, and *n* is the number of soil parameters.

### 4.5. Statistical Data Analyses

Statistical tests for the effects of forest ages variation on soil nutrient indicators were performed. A two–way analysis of variance (ANOVA) and Duncan’s multiple comparison test were used to analyze the statistical differences between the age groups and the soil layers at the *p* < 0.05 level. The original data were log–transformed to satisfy the normality and homoscedasticity assumptions of ANOVA. Microsoft Excel 2016 and SPSS 25.0 for Windows were used for statistical data analyses. The SEM was performed using AMOS 24.0 for Windows.

## 5. Conclusions

The six soil nutrient indicators including SOM, TN, TP, AN, AP, and AK in five age groups of Chinese fir plantations were measured, and a structure equation model (SEM) was built based the measurement data in subtropical China. The SFI values of Chinese fir forests were calculated based on the weights of the SEM in our study. We also found that the SOM, soil TN, and AN content were significantly different in the five age groups and among the soil layers in our study. The soil TN, AP, and AK are the most important nutrient indicators in evaluating SFI in Chinese fir forest ecosystem. Overall, the highest SFI was found in the over-matured forests among the age group stands. The SFI was significantly higher in the topsoil than in the deeper soils with high soil nutrient indicators among the soil depth. The SEM for evaluating the soil nutrient status and calculating weight of SFI in our research provides an advanced technology and reliable method for evaluating soil fertility in forest ecosystems.

## Figures and Tables

**Table 1:** The nutrient contents of soils (from 0 to 60 cm) in each age group of Chinese fir forest stands. Note: lower case letters indicate significant differences (*p* < 0.05).

Age Group	SOM	TN	TP	AN	AP	AK
	(g·kg^−1^)	(g·kg^−1^)	(g·kg^−1^)	(mg·kg^−1^)	(mg·kg^−1^)	(mg·kg^−1^)
I	14.29 ± 0.37 d	0.81 ± 0.08 ab	0.19 ± 0.07	60.98 ± 6.26	3.68 ± 2.78	40.16 ± 14.11
II	13.07 ± 0.15 e	0.74 ± 0.05 b	0.17 ± 0.05	57.44 ± 11.83	2.78 ± 1.90	44.26 ± 12.57
III	15.15 ± 0.41 c	0.82 ± 0.05 ab	0.19 ± 0.07	61.71 ± 12.48	2.60 ± 1.50	46.87 ± 13.17
IV	16.45 ± 0.27 b	0.89 ± 0.09 a	0.18 ± 0.05	65.20 ± 11.76	5.31 ± 6.82	49.97 ± 14.87
V	18.15 ± 0.58 a	0.86 ± 0.03 a	0.20 ± 0.05	70.90 ± 10.75	5.01 ± 7.78	54.19 ± 10.94

**Table 2:** Modified model fitness metrics for assessing the degree of adaptation for each indicator.

Model Fitting Index	Statistical Value	Reference Value	Degree of Adaptation
CMIN/DF	2.844	1–3	Fit
CFI	0.970	>0.90	Fit
TLI	0.911	>0.90	Fit
IFI	0.971	>0.90	Fit
SRMR	0.0536	<0.08	Fit

**Table 3:** The weights for evaluating the soil fertility index.

Target Layer	Code Level	Weighting	Indicator Layer	Weighting
Soil fertility	Total nutrients	0.4604	SOM	0.3855
			TN	0.4154
			TP	0.1991
	Available nutrients	0.5396	AN	0.2138
			AP	0.3941
			AK	0.3921

**Table 4:** The environmental characteristics of the soil sampling sites.

Age Group	Age	Slope Direction	Slope	Average Height	Average DBH	Stand Density	Undergrowth
	(years)		(°)	(m)	(cm)	(plants·ha^−1^)	(% cover)
Young forest	6	Northeast	23	7.1	9.3	3012	47
Middle-aged forest	12	East	26	8.7	10.8	3298	15
Near-mature forest	18	East	31	12.9	14.6	1321	62
Mature forest	24	Northeast	29	14.1	17.8	1203	75
Over-mature forest	31	East	35	29.8	30.2	1017	93

## Data Availability

Not applicable.
